# Supplementary material for: The Antibiotic Treatment of Calf Diarrhea in Four European Countries: A Survey
Source: Antibiotics (Basel). 2021 Jul 26;10(8):910. doi: 10.3390/antibiotics10080910 (PMC8388724; doi:10.3390/antibiotics10080910)
Supplement: Supplementary file 1 [file antibiotics-10-00910-s001.zip › antibiotics-1293869-supplementary.pdf]

antibiotics

MDPI

Article

The Antibiotic Treatment of Calf Diarrhea in Four European Countries: A Survey

Cassandra Eibl [1], Ricardo Bexiga [2], Lorenzo Viora [3], Hugues Guyot [4], José Félix [2], Johanna Wilms [5], Alexander Tichy [6] and Alexandra Hund [1,7,*]

1 University Clinic for Ruminants, Department for Farm Animals and Veterinary Public Health, University of Veterinary Medicine Vienna, 1210 Vienna, Austria; Cassandra.Eibl@vetmeduni.ac.at
2 Centro de Investigação Interdisciplinar em Sanidade Animal, Faculdade de Medicina Veterinária, Universidade de Lisboa, 1300-477 Lisbon, Portugal; ricardo-bexiga@fmv.ulisboa.pt (R.B.); jose_duarte_felix@hotmail.com (J.F.)
3 School of Veterinary Medicine, College of Medical, Veterinary and Life Sciences, University of Glasgow, G611GH Glasgow, UK; Lorenzo.Viora@glasgow.ac.uk
4 Clinical Department of Production Animals, Fundamental and Applied Research for Animals and Health, University of Veterinary Medicine, 1210 Vienna, Austria; Hugues.Guyot@uliege.be
5 Tierarztpraxis Geisenhausen, 84144 Geisenhausen, Germany; johanna.wilms@gmx.de
6 Platform Bioinformatics and Biostatistics, Department for Biomedical Sciences, University of Veterinary Medicine, 1210 Vienna, Austria; Alexander.Tichy@vetmeduni.ac.at
7 Agricultural Center for Cattle, Grassland, Dairy, Game and Fisheries of Baden-Württemberg (LAZBW), 88326 Aulendorf, Germany
* Correspondence: Alexandra.Hund@lazbw.bwl.de

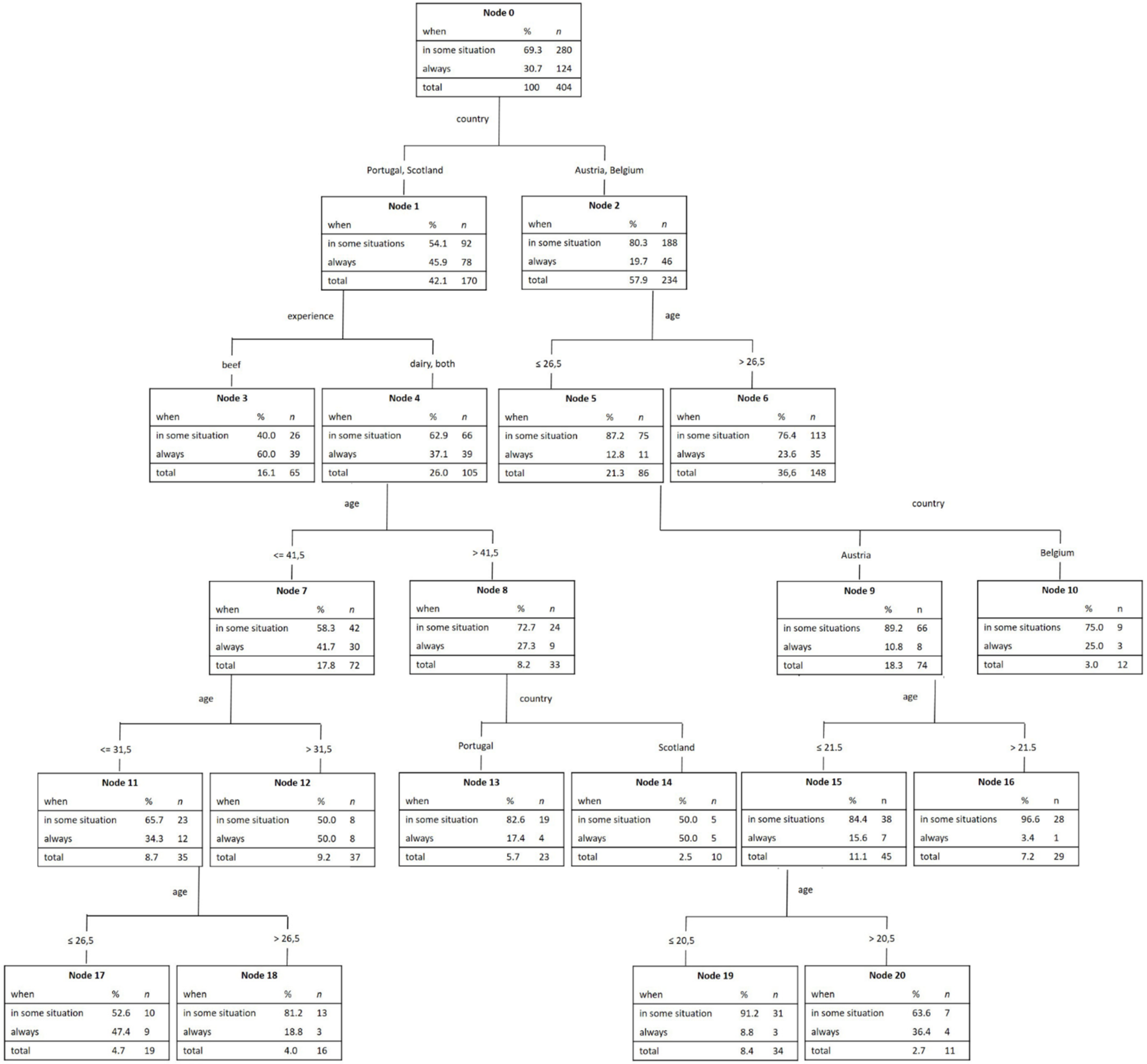
Node 0
when % n
in some situation 69.3 280
always 30.7 124
total 100 404
country
Portugal, Scotland
Austria, Belgium
Node 1
when % n
in some situations 54.1 92
always 45.9 78
total 42.1 170
Node 2
when % n
in some situation 80.3 188
always 19.7 46
total 57.9 234
experience
age
beef
dairy, both
≤ 26,5
> 26,5
Node 3
when % n
in some situation 40.0 26
always 60.0 39
total 16.1 65
Node 4
when % n
in some situation 62.9 66
always 37.1 39
total 26.0 105
Node 5
when % n
in some situation 87.2 75
always 12.8 11
total 21.3 86
Node 6
when % n
in some situation 76.4 113
always 23.6 35
total 36,6 148
age
country
<= 41,5
> 41,5
Austria
Belgium
Node 7
when % n
in some situation 58.3 42
always 41.7 30
total 17.8 72
Node 8
when % n
in some situation 72.7 24
always 27.3 9
total 8.2 33
Node 9
% n
in some situations 89.2 66
always 10.8 8
total 18.3 74
Node 10
% n
in some situations 75.0 9
always 25.0 3
total 3.0 12
age
country
age
<= 31,5
> 31,5
Portugal
Scotland
≤ 21.5
> 21.5
Node 11
when % n
in some situation 65.7 23
always 34.3 12
total 8.7 35
Node 12
when % n
in some situation 50.0 8
always 50.0 8
total 9.2 37
Node 13
when % n
in some situation 82.6 19
always 17.4 4
total 5.7 23
Node 14
when % n
in some situation 50.0 5
always 50.0 5
total 2.5 10
Node 15
% n
in some situations 84.4 38
always 15.6 7
total 11.1 45
Node 16
% n
in some situations 96.6 28
always 3.4 1
total 7.2 29
age
age
≤ 26,5
> 26,5
≤ 20,5
> 20,5
Node 17
when % n
in some situation 52.6 10
always 47.4 9
total 4.7 19
Node 18
when % n
in some situation 81.2 13
always 18.8 3
total 4.0 16
Node 19
when % n
in some situation 91.2 31
always 8.8 3
total 8.4 34
Node 20
when % n
in some situation 63.6 7
always 36.4 4
total 2.7 11

Figure S1. CART: Association of different factors on the use of antibiotics according to the question “If you usually use antimicrobials please state when”.
